# Osteogenic Differentiation of Human Adipose-Derived Stem Cells Seeded on a Biomimetic Spongiosa-like Scaffold: Bone Morphogenetic Protein-2 Delivery by Overexpressing Fascia

**DOI:** 10.3390/ijms23052712

**Published:** 2022-02-28

**Authors:** Bin Ren, Oliver B. Betz, Daniel Seitz, Christian Thirion, Michael Salomon, Volkmar Jansson, Peter E. Müller, Volker M. Betz

**Affiliations:** 1 Department of Orthopedic Surgery, Physical Medicine and Rehabilitation, University Hospital Munich, Campus Grosshadern, Ludwig-Maximilians-University, Munich, Marchioninistr. 15, 81377 Munich, Germany; oliver.b.betz@gmail.com (O.B.B.); volkmar.jansson@med.uni-muenchen.de (V.J.); peter.mueller@med.uni-muenchen.de (P.E.M.); volker.betz@yahoo.com (V.M.B.); 2 Departments of Orthopedics, Zhongnan Hospital of Wuhan University, 430072 Wuhan, China; 3 Department of Chemical Engineering, Massachusetts Institute of Technology, 77 Massachusetts Avenue, Cambridge, MA 02139, USA; 4 BioMed Center Innovation gGmbH, 95448 Bayreuth, Germany; dseitz@biomed-center.com; 5 Sirion Biotech GmbH, Am Klopferspitz 19, 82152 Martinsried, Germany; thirion@sirion-biotech.de (C.T.); salomon@sirion-biotech.de (M.S.)

**Keywords:** adipose-derived stem cells, fascia, osteogenic differentiation, delivery system, scaffold

## Abstract

Human adipose-derived stem cells (hADSCs) have the capacity for osteogenic differentiation and, in combination with suitable biomaterials and growth factors, the regeneration of bone defects. In order to differentiate hADSCs into the osteogenic lineage, bone morphogenetic proteins (BMPs) have been proven to be highly effective, especially when expressed locally by route of gene transfer, providing a constant stimulus over an extended period of time. However, the creation of genetically modified hADSCs is laborious and time-consuming, which hinders clinical translation of the approach. Instead, expedited single-surgery gene therapy strategies must be developed. Therefore, in an in vitro experiment, we evaluated a novel growth factor delivery system, comprising adenoviral BMP-2 transduced fascia tissue in terms of BMP-2 release kinetics and osteogenic effects, on hADSCs seeded on an innovative biomimetic spongiosa-like scaffold. As compared to direct BMP-2 transduction of hADSCs or addition of recombinant BMP-2, overexpressing fascia provided a more uniform, constant level of BMP-2 over 30 days. Despite considerably higher BMP-2 peak levels in the comparison groups, delivery by overexpressing fascia led to a strong osteogenic response of hADSCs. The use of BMP-2 transduced fascia in combination with hADSCs may evolve into an expedited single-surgery gene transfer approach to bone repair.

## 1. Introduction

Trauma and bone disease frequently result in fractures or large critical bone defects, and their management often necessitates the substitution of bone. Traditional therapies include autograft and allograft transplantation, of which autologous bone transplantation is recognized as the clinical “gold standard” since all the essential requirements for bone regeneration in terms of osteoconduction, osteoinduction and osteogenesis are satisfied [1,2]. However, in addition to the limited availability of bone autograft, chronic pain and donor site morbidity limit the effectiveness of this option. Although allograft is widely available, the problems of resorption, immune reaction and disease transmission restrict its application [3,4]. Attempts have been made to identify effective alternative methods, but there is still no consensus on an ideal therapy. To date, available osseous substitutes fall short of achieving a robust and reliable bone formation clinically.

When utilizing stem cells for the colonization of an implant, a critical question is how to trigger their differentiation towards a specific cell type [5,6]. Growth factors are crucial signaling molecules that regulate cell migration, proliferation, differentiation and extracellular matrix (ECM) secretion, which are key components for tissue regeneration. These molecules can be secreted and transported to the injured area to enable spontaneous tissue restoration. However, due to the insufficient concentration and quantity of these molecules, as well as the lack of a “bridge” to generate new tissues between fracture ends, large defects are not easy to repair. To compensate for these limitations, tissue engineering technologies integrating stem cells, growth factors and scaffolds have been introduced into research and clinical fields [7]. However, as the key component of tissue regeneration, growth factors are highly sensitive to temperature, pH and environment [8], and their rapid degradation characteristics further challenge their practical application [9].

In initial strategies, massive amounts of exogenous molecule proteins were applied directly to tissue defect sites, risking potential heterotopic ossification and even cancer formation, also bringing heavy economic burdens to patients [10]. Afterwards, a variety of attempts were made in order to achieve sustained release of growth factors to bone defects. Delivery systems from scaffold materials have been developed to provide long-term bioactivity of growth factors. These systems incorporate molecule proteins on the surfaces or within scaffold materials by physicochemical means (e.g., encapsulated in microspheres, hydrogen bonding or hydrophobic interactions) [11,12,13]. However, toxic reagents, harsh organic solvents and complex fabrication procedures still remain obstacles to clinical application [14]. Alternatively, gene transfer technology would allow the genetically modified stem cells to continuously secrete growth factors while maintaining the capacity of osseous differentiation.

Numerous studies have demonstrated that bone formation can be induced by the implantation of gene-modified stem cells or progenitor cells [15,16]. In particular, adipose tissue represents an appealing source of cells useful for regenerative medicine, since its harvest is relatively easy to perform and is associated with very little donor site morbidity [17,18]. Hence, human adipose-derived stem cells (hADSCs) have been investigated extensively with respect to their ability for osteogenic differentiation [18,19]. Moreover, scientists working in the field of gene therapy have proven that bone morphogenetic protein-2- (BMP-2) transduced hADSCs can regenerate large bone defects in pre-clinical in vivo models [20,21]. However, while innovative devices allow rapid harvest and isolation of hADSCs [22], the additional genetic modification of isolated hADSCs with high efficiency cannot be achieved within the short duration of a single surgery. Moreover, genetically modified hADSCs distributed on a scaffold just briefly prior to implantation are more likely to become dislocated from the biomaterial and may potentially lead to heterotopic bone formation.

Therefore, in order to develop safe, expedited, single-surgery gene therapy approaches, our group has been working with gene activated autologous tissue grafts [23,24,25,26,27,28,29,30,31,32,33]. The genetic modification of such tissue grafts can be accomplished within one hour or less, enabling surgeons to re-implant the grafts within the same surgical procedure.

In previous studies, we have discovered that fascia tissue fragments can be readily transduced with adenoviral vectors, leading to BMP-2 expression with nanogram quantities for at least 90 days in vitro. Transduction efficiency was even higher than in fragments of muscle tissue without fascia or adipose tissue fragments [31,33].

Fascia tissue represents thin layers of connective tissue covering organs and tissue structures. Fascia tissue is ubiquitously distributed in the body, containing fibroblasts and other cell types embedded within a network of collagen fibers [34,35,36].

The goal of the present study was to evaluate the capability of genetically modified fascia tissue to serve as a delivery system for BMP-2inducing osteogenesis in hADSCs seeded on a biomimetic spongiosa-like scaffold. To achieve this, we utilized an in vitro co-culture system and investigated the characteristics of BMP-2 release from fascia and its osteoinductive effects in comparison to direct transduction of hADSCs and supplementation with recombinant BMP-2. As a prelude to future in vivo studies, the present in vitro evaluation provides information about the feasibility of using transduced fascia as a growth factor delivery system and supports the development of an expedited gene-based bone repair strategy involving hADSC-seeded scaffolds.

## 2. Results

### 2.1. Monolayer Experiment

#### 2.1.1. Adenoviral Transduction Efficiency of hADSCs in Monolayer Culture

**Figure 1:**
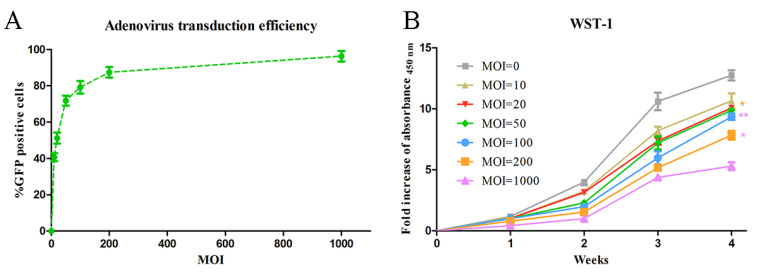
Transduction efficiency and proliferation of hADSCs transduced with adenovirus: (**A**) Summary result of adenoviral transduction efficiency using different MOI. (**B**) WST-1 measurement of hADSCs transduced with adenovirus using different MOI. Values given represent means ± SE, * for *p* < 0.05, and ** for *p* < 0.01.

To explore the adenovirus transduction efficiency in human adipose-derived stem cells (hADSCs) using different multiplicities of infection (MOI), hADSCs were transduced by adenovirus-encoding green fluorescent protein (Ad.GFP) with MOI of 10, 20, 50, 100, 200 and 1000 at passage 4, and control cells were left non-transduced. Transduction efficiency was evaluated 72 h post-transduction by Fluorescence-Activated Cell Sorting (FACS). During FACS evaluation, live cells could be chosen using gating tools as the main cell population. Green, fluorescent protein-labeled cells were subsequently sorted from the main cell population after an electronic charge, and transduction efficiency was determined as the fraction of the sorted subpopulation within the main cell population (Figure 1A).

Overall, infection with adenovirus vectors mediated extensive transduction of hADSCs. As shown in Figure 1A, adenovirus transduction was very efficient. Even a very low MOI of 10 led to a transduction efficiency of 40%. By increasing the MOI from 10 to 1000, the percentage of transduced hADSCs rose from around 40% to 95%. The MOI of 100 was already sufficient to infect around 80% of hADSCs. Transduction with MOI of 100 and higher led to a transduction of the vast majority of hADSCs, which was significantly higher than with MOI of 10, 20 and 50. No significant difference was found between MOI of 100, 200 and 1000.

#### 2.1.2. Proliferation Test of Adenovirus-Transduced hADSCs

In order to determine whether transduction with adenovirus influenced proliferation of hADSCs, proliferation was analyzed over a period of 4 weeks using a WST-1 assay. After 1 week of incubation, proliferation increased significantly in all experimental groups, with a further significant increase after 2 weeks. Comparatively, the proliferation of hADSCs infected at MOI of 10, 20, 50 and 100 was lower, but not significantly different from the uninfected control, suggesting that adenovirus infection did not have a significant suppressive effect on cell proliferation under the aforementioned transduction conditions. However, transduction at higher MOI (200 and 1000) significantly inhibited cell proliferation compared with infection at lower MOI and the non-transduced control, indicating an inhibiting effect on proliferation when transduction was performed with high vector doses. MOI of 1000, the highest MOI applied in this experiment, exhibited the lowest cell proliferation. This was significantly lower than all other groups (Figure 1B).

#### 2.1.3. Calcium Depositions of Ad.hBMP-2-Transduced hADSCs in Monolayer Culture

**Figure 2:**
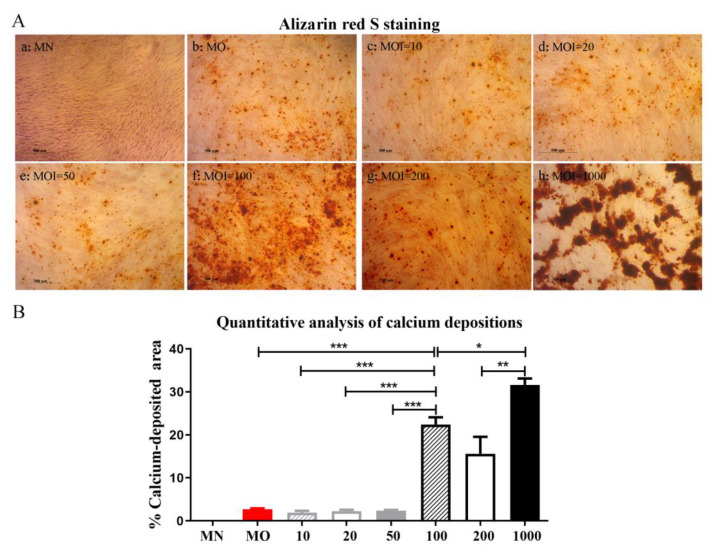
Analysis of calcium deposition within Ad.hBMP-2-transduced hADSCs using different MOI after 4 weeks in monolayer culture: (**A**): Micrographs, Alizarin red S staining. (**a**) MN; (**b**) MO; (**c**) MOI = 10; (**d**) MOI = 20; (**e**) MOI = 50; (**f**) MOI = 100; (**g**) MOI = 200; (**h**) MOI = 1000. Scale bar = 1 mm. (**B**) Morphometric analysis. Calcium was not detected in the MN group. hADSCs transduced with MOI of 100 and 1000 deposited significantly more calcium than the other groups. Most calcium depositions were observed in the group transduced with an MOI of 1000. (* for *p* < 0.05, ** for *p* < 0.01 and *** for *p* < 0.001).

To further evaluate the osteogenic differentiation capacity of Ad.hBMP-2-transduced hADSCs using different MOI, Alizarin red S mineralization analysis was performed after 4 weeks of incubation. Non-transduced hADSCs cultured in osteogenic medium (MO) and normal growth medium (MN) were also analyzed as controls. Alizarin bright red color staining of osteoblastic calcium deposition was analyzed qualitatively (Figure 2A) and quantitatively (Figure 2B) by measurement of calcium-deposited area.

After 4 weeks of incubation, the seeded hADSCs in 24-well plates were distributed evenly and almost reached 100% confluency in most groups. Comparatively, in the group of MOI = 1000, cells gathered together in clusters. Matrix deposits (black spots) secreted by differentiated stem cells could be seen in all transduction groups and in the non-transduced MO group before staining, and were further confirmed by Alizarin red S staining (Figure 2A). The observed mineralization was MOI-dependent. Collectively, transduction under MOI = 100 and MOI = 1000 induced robust osteoblastic differentiation as shown by calcium deposits, which was significantly higher than in all other groups. MOI = 1000 induced the largest amounts of calcium deposits. On the contrary, hADSCs in transduction groups of MOI = 10, 20 or 50 did not show any significant enhancement of mineralization in response to secreted BMP-2 stimulus (Figure 2B).

#### 2.1.4. Adenoviral Transduction of Fascia Tissue

**Figure 3:**
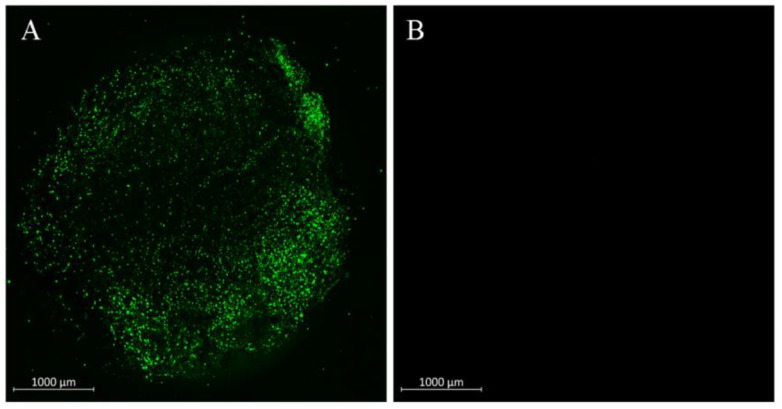
Adenoviral modification of 4 mm muscle/fascia fragments under a laser-scanning confocal microscope. (**A**): Green fluorescent signals were detected in cells residing on the surface of adenovirus-encoding GFP- (Ad.GFP) modified fascia tissue using 4 × 10^8^ pfu. (**B**): Green fluorescent signals were absent in non-transduced fascia tissue.

To analyze gene transduction efficiency of adenovirus to fascia tissue, each muscle/fascia tissue fragment was transduced with 4 × 10^8^ pfu of adenoviral vectorencoding GFP (Ad.GFP). As indicated in Figure 3A, cells residing on the surface of the fascia tissue were effectively transduced by adenovirus, and considerable green, fluorescent signals were detected in AdGFP-transduced tissues. No green signals were detected in non-transduced control tissues (Figure 3B).

### 2.2. Three-Dimensional Scaffold Experiment

#### 2.2.1. Comparison of Different hBMP-2 Delivery Approaches

It has been shown that bone morphogenetic proteins (BMPs) have a powerful capacity to initiate new bone formation. Therefore, we compared the osteogenic differentiation capacity of hADSCs seeded on 3D scaffolds utilizing different approaches for local delivery of hBMP-2. MOI of 100 were chosen for the transduction of hADSCs with Ad.hBMP-2, based on the results of the monolayer experiment with respect transduction efficiency, cell proliferation and osteogenic response. For transduction of fascia tissue, 4 × 10^8^ pfu of the adenoviral BMP-2 vector was used. This vector dose has been identified as the optimal dose for fascia tissue transduction in previous experiments.

**Figure 4:**
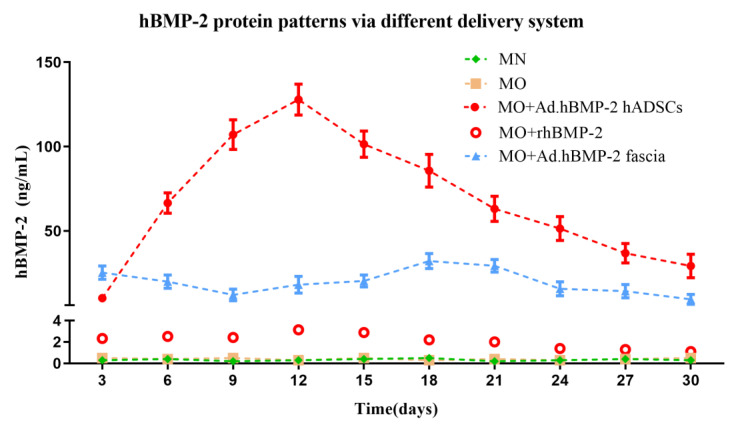
Human BMP-2 protein level patterns via different hBMP-2 delivery systems measured in supernatants harvested every 3 days until day 30. Human BMP-2 was continuously released from group MO + Ad.hBMP-2 hADSC and group MO + Ad.hBMP-2 fascia. In the MO + rhBMP-2 group, the same amounts of recombinant proteins as those produced by the MO + Ad.hBMP-2 hADSC group were added, and degraded significantly to a minimal level (around 2 ng/mL) after 3 days in culture at each time point (red circle dot, without connected line). Little to no hBMP-2 was detected in non-transduced groups: MN and MO.

BMP-2 was continuously synthesized by transduced hADSCs during the whole culture period, peaking at day 12 at about 130 ng/mL BMP-2, and declined gradually by 15–30 days of culture. In the non-transduced group, the same BMP-2 concentrations as the BMP-2 synthesis level were achieved by supplementing rhBMP-2 proteins in culture medium. Interestingly, after 3 days incubation, the added rhBMP-2 proteins were found to degrade to a very low level (about 2 ng/mL) at each time point. In a series of experiments, hBMP-2 levels in the Ad.hBMP-2-transduced fascia group measured 25.29 ng/mL at day 3 and then fluctuated between 10 ng/mL and 30 ng/mL throughout the culture period. Non-transduced cells did not show any detectable BMP-2 release into the medium (Figure 4).

#### 2.2.2. Evaluation of Cell Distribution within the Scaffold

**Figure 5:**
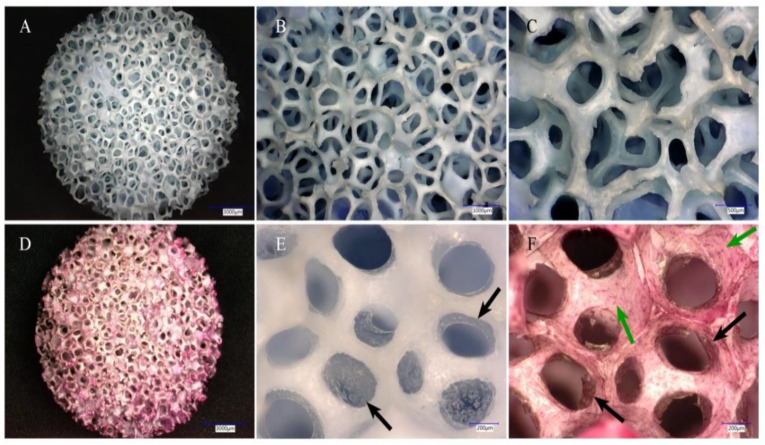
The morphological structure of the untreated scaffold (**A**–**C**) and representative 3D digital micrographs of hADSC-colonized scaffold cultured for 28 days (**D**–**F**). (A × 20, B × 50, C × 100): The 3D digital microscope images show the scaffold’s 3-D, highly porous structure with ordered interconnected pore channels. (D × 20): Haematoxylin and Eosin (H&E) staining of hADSC-seeded 3D scaffold: after 28 days of incubation, the entire hADSC-colonized scaffold was thoroughly stained to red and blue with H&E. (E × 200): It is exhibited that the pores were averagely distributed with stem cells (black arrows). (F × 200): H&E-stained hADSCs located in the pores (black arrows) and on the scaffold wall surface (green arrows) could still be clearly observed, even though a large fraction of cells was damaged during the process of staining. E and F were taken in the same position of the scaffold.

It is critical that a scaffold promotes cell adhesion and proliferation. Therefore, cell distribution and growth capacity on the scaffold were evaluated using a 3D digital microscope after Hematoxylin and Eosin (H&E) staining. As shown in Figure 5, no visible scaffold degradation was observed over the 4 weeks of culture. After 4 weeks of incubation, the cells proliferated layer upon layer and connected with other cells. Most of the pores were covered by proliferated cells (black arrows), which appeared similar to paved blankets. After staining with H&E, cells growing in the pores (black arrows) as well as the scaffold walls (green arrows) could be clearly observed, even though a large fraction of cells was damaged during staining. In summary, cells seeded on the 3D scaffold were not only homogeneously distributed on the surface of scaffold porous walls, but also migrated through the interconnected pores and were found distributed in deeper areas of the scaffold. However, we did not confirm this by microscopy for the innermost parts of the scaffolds.

#### 2.2.3. Calcification within Ad.hBMP-2-Transduced Muscle/Fascia Fragments

**Figure 6:**
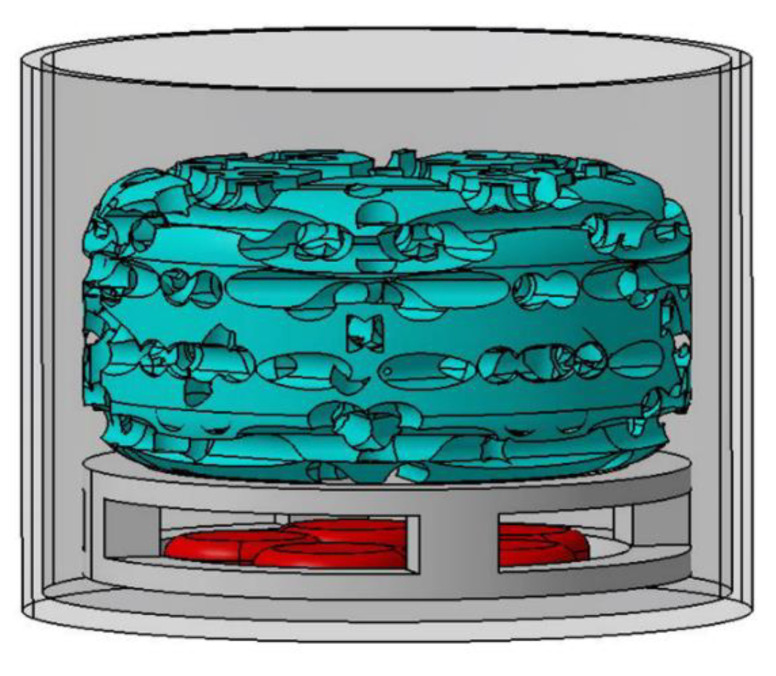
Designed scaffold and muscle/fascia tissue fragments co-culture system: circular plastic bracket designed to support the scaffold and avoid direct contact with the underlying muscle/fascia fragments; co-culture system in 12-well plates.

Ad.hBMP-2-transduced muscle/fascia tissue fragments were co-cultured together with hADSCs seeded on 3D scaffolds in order to deliver hBMP-2, inducing an osteogenic response (Figure 6).

**Figure 7:**
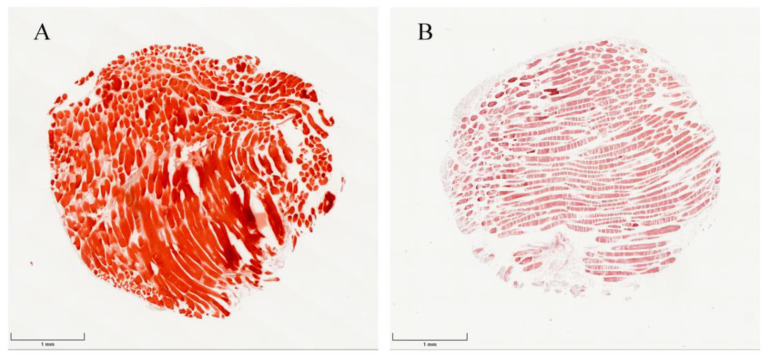
Alizarin red S stained 4 mm muscle/fascia tissue fragments: (**A**) Ad.hBMP-2-transduced muscle/fascia co-cultured together with the scaffold for 4 weeks; MO + Ad.hBMP-2 fascia group: calcium depositions were detected through the entire Ad.hBMP-2-transduced muscle/fascia tissue, which were stained a bright red color by Alizarin red S. (**B**) Untreated fresh muscle/fascia tissue: no calcification was observed in untreated muscle/fascia tissue. Scale bar = 1 mm.

Calcium deposits within the co-cultured muscle/fascia tissue fragments were identified by Alizarin red S staining (Figure 7). After 4 weeks of incubation, the entire muscle/fascia tissue fragments were detected with Alizarin red S-positive stained calcium deposition (Figure 7A), while calcification was undetectable in untreated fresh muscle/fascia tissue fragments (Figure 7B).

#### 2.2.4. Alkaline Phosphatase (ALP) Activities

**Figure 8:**
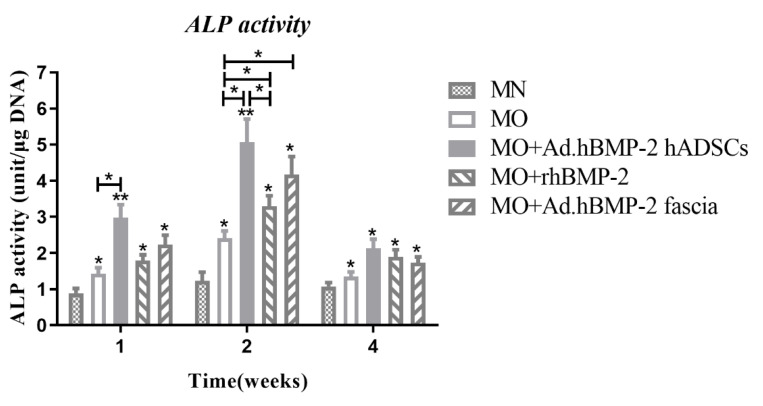
Alkaline phosphatase (ALP) activity analysis at 1 week, 2 weeks and 4 weeks. The significance level of the four MO groups vs. the MN group was marked on the top of column without capped line (* for *p* < 0.05, and ** for *p* < 0.01).

ALP activities at 1 week were significantly higher for the BMP-2-treated groups than for the MN and MO groups and kept increasing from 1 week to 2 weeks (Figure 8). In all groups, ALP activity decreased by week 4. Among BMP-2-treated groups, the BMP-2-transduced hADSC group elicited the highest mean values for ALP activity. However, no statistically significant difference was noted as compared to BMP-2 transduced fascia. At week 2, ALP activity was significantly higher for the BMP-2-transduced hADSC group than for the recombinant BMP-2 group. At week 4, no significant differences in ALP activity were observed among the BMP-2-treated groups or MO group (Figure 8).

#### 2.2.5. Gene Expression Analysis

**Figure 9:**
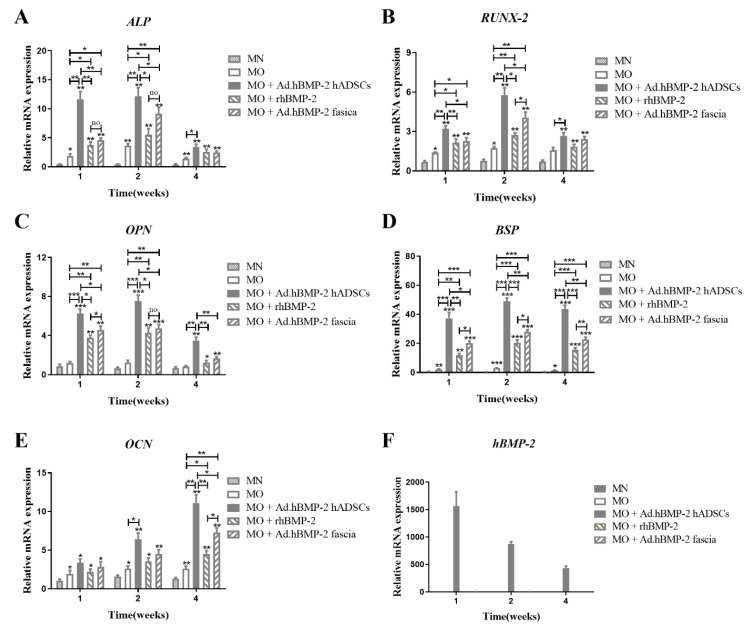
Quantitative RT-PCR analysis of osteogenic gene expression at 1 week, 2 weeks and 4 weeks in hADSCs seeded on scaffolds treated by different hBMP-2 delivery modes. (**A**) *ALP*; (**B**) *RUNX-2*; (**C**) *OPN*; (**D**) *BSP*; (**E**) *OCN*; (**F**) h*BMP-2*. Osteogenic marker genes (**A**–**E**) were significantly up-regulated in all the hBMP-2 treated groups. Comparatively, the MO + Ad.hBMP-2 hADSC group presented the highest levels of osteogenic gene expression, followed by the MO + Ad.hBMP-2 fascia group and MO + rhBMP-2 group. Human *BMP-2* was only detected in the MO + Ad.hBMP-2 hADSC group. The significance level of the four MO vs. MN groups was marked on the top of column without capped line (* for *p* < 0.05, ** for *p* < 0.01 and *** for *p* < 0.001).

The osteogenic response of hADSCs seeded on scaffolds was evaluated by qRT-PCR analysis of *ALP*, *RUNX-2*, *OPN*, *BSP* and *OCN* expression (Figure 9). All the hBMP-2-treated groups expressed *ALP*, *RUNX-2*, *OPN*, *BSP* and *OCN* at levels that were significantly higher than those of the untreated MO and MN groups at most time points, while the MO group showed a significantly higher level than the MN group. During the first two weeks, the early stage of the culture period, *ALP* and *RUNX-2* mRNA expressions noticeably increased in the hBMP-2 group and Ad.hBMP-2-transduced fascia group, whereas the expression levels even further increased in the Ad.hBMP-2-transduced hADSC group, resulting in significantly higher expressions than in the hBMP-2 protein group and Ad.hBMP-2-transduced fascia group. Comparatively, the Ad.hBMP-2-transduced fascia group expressed significantly higher *ALP* and *RUNX-2* mRNA levels than the hBMP-2 protein group at week 2 (Figure 9A,B). *OPN* and *BSP* mRNA expressions raised in all three BMP-2-treated groups throughout the culture period and peaked at week 2. The expression levels for *OPN* and *BSP* within the Ad.hBMP-2-transduced hADSC group increased significantly more than those observed in the hBMP-2 protein group and the Ad.hBMP-2-transduced fascia group. Moreover, the Ad.hBMP-2-transduced fascia group expressed significantly higher *OPN* than the hBMP-2 group at weeks 1 and 2, and *BSP* throughout the whole culture period (Figure 9C,D). *OCN* in the MN group was expressed at the lowest level, whereas the expression level in the MO group significantly increased but still remained at a relatively low level. *OCN* expression in the Ad.hBMP-2-transduced hADSC group, hBMP-2 protein group and Ad.hBMP-2-transduced fascia group raised significantly in weeks 4 to levels that were higher than in the MO control. The mRNA expression level of OCN in the Ad.hBMP-2-transduced hADSC group increased significantly more than that in the hBMP-2 protein group and Ad.hBMP-2-transduced fascia group, while a higher expression level was observed in the Ad.hBMP-2-transduced fascia group than in the hBMP-2 protein group (Figure 9E). The transduction with Ad.hBMP-2 induced a 1500-fold overexpression of *hBMP-2* in hADSCs at 1 week, which dropped to levels of 800-fold in week 2 and 500-fold in week 4 as compared to that of non-transduced hADSCs. In the hBMP-2 protein group and the Ad.hBMP-2-transduced fascia group, hADSCs did not show any detectable *hBMP-2* mRNA overexpression throughout the entire culture period (Figure 9F).

#### 2.2.6. Immunofluorescent Staining and Quantitative Analysis

**Figure 10:**
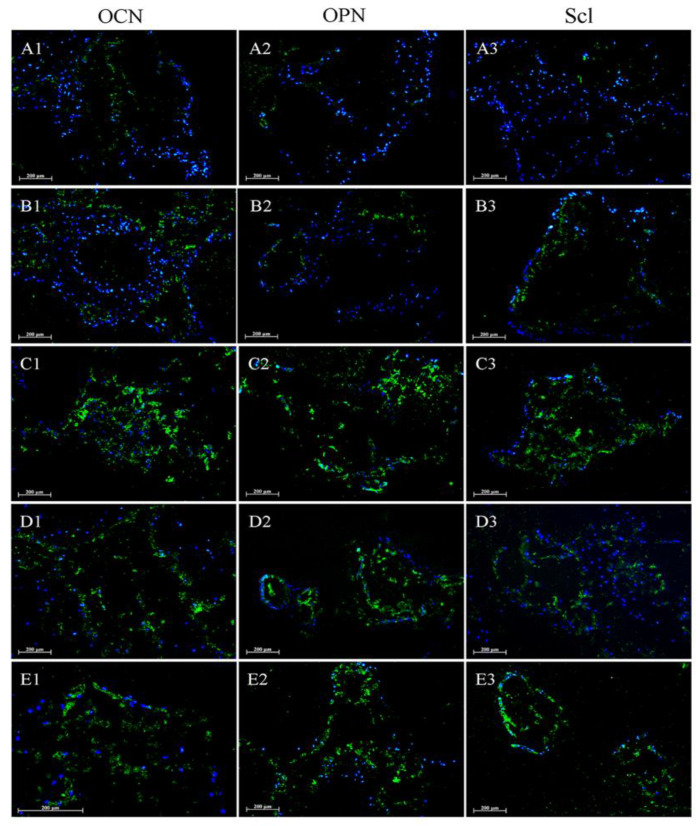
Immunofluorescent staining of hADSC-seeded scaffolds treated by different hBMP-2 delivery modes after 4 weeks of incubation. Samples treated with MN (**A1**–**A3**); MO (**B1**–**B3**); MO + Ad.hBMP-2 hADSC (**C1**–**C3**); MO + rhBMP-2 (**D1**–**D3**); MO + Ad.hBMP-2 fascia (**E1**–**E3**). Immunofluorescent staining products of OCN, OPN and Scl are presented in green; nuclei of cells stained with DAPI are presented in blue. Scale bar = 200 μm.

After four weeks of incubation, the osteogenic response within the hADSC-seeded scaffolds was investigated by immunofluorescence detection of OCN, OPN and Scl. Immunofluorescent staining for OCN, OPN and Scl appears green and nuclei are stained blue with DAPI. Cells settled at the surfaces of the scaffold lattice and filled the void areas after 4 weeks, covering the scaffold with a continuous layer, which is consistent with the results of H&E staining. When non-transduced hADSC-seeded scaffolds were cultured in normal growth medium (MN), OCN, OPN and Scl were almost undetectable (Figure 10A1–A3). When cultured in osteogenic medium (MO), OCN, OPN and Scl were positively stained, although at very low levels (Figure 10B1–B3). Comparatively, all the BMP-2-treated groups, the MO + Ad.hBMP-2 hADSC group (Figure 10C1–C3), MO + rhBMP-2 group (Figure 10D1–D3) and MO + Ad.hBMP-2 fascia group (Figure 10E1–E3) showed increased OCN, OPN and Scl expression compared to the MN and MO groups.

**Figure 11:**
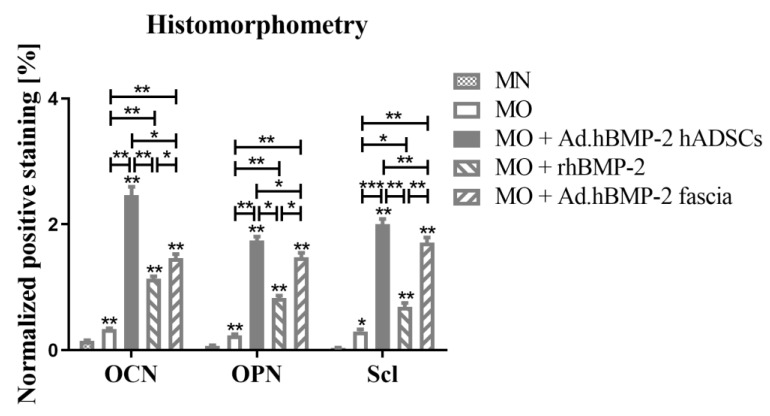
Quantitative analysis of the positively stained area. The quantified products of OCN, OPN and Scl were normalized to the corresponding cell numbers in the same microscopic field. The significance level of MN vs. the four MO groups was marked on the top of the column without a capped line (* for *p* < 0.05, ** for *p* < 0.01 and *** for *p* < 0.001).

The quantitative analysis (Figure 11) indicated that OCN, OPN and Scl expressions in normal growth medium-cultured hADSC-seeded scaffolds (MN group) were almost negative. The expression levels in the same BMP-2 untreated hADSC-seeded scaffolds were significantly enhanced by osteogenic medium (MO group) as compared to normal growth medium, however, these were still significantly lower than in BMP-2-treated groups. Of the three BMP-2-treated groups, the MO + Ad.hBMP-2 hADSC group expressed the highest levels of OCN, OPN and Scl, followed by the MO + Ad.hBMP-2 fascia group and the MO + rhBMP-2 group.

## 3. Discussion

In an effort to develop novel, cost-effective, single-surgery gene therapy approaches to bone regeneration, our group has been focusing on the work with gene-activated tissue grafts [16,37]. Fragments of muscle and adipose tissue transduced to overexpress BMP-2 or BMP-7 have been utilized to repair bone defects in rodents with promising results [23,24,26,27]. Moreover, we have recently reported that fascia tissue can be transduced with higher efficiency than muscle or adipose tissue, resulting in high-level transgene expression over an extended period of time and osteoinduction within the muscle/fascia fragment [31,33]. While autologous muscle and adipose tissue fragments can be used to fill smaller bone defects, fascia is just a thin tissue layer lacking a voluminous three-dimensional character. However, in the case of large osseous defects, transduced fascia may be applied in combination with cell-seeded artificial scaffolds. Hence, the goal of the present study was to evaluate the ability of fascia tissue to deliver BMP-2 to hADSCs seeded on highly porous scaffolds in a sustained fashion, providing sufficient osteogenic stimulus. Delivery by overexpressing fascia was compared to direct transduction of hADSCs and supplementation with recombinant BMP-2.

In the first step of this study, we established the optimal MOI using adenoviral vectors for the employed hADSCs in order to achieve high-level BMP-2 expression, but without hampering the proliferation and viability of hADSCs due to cytotoxicity of the adenoviral vectors [38]. An MOI of 100 was found to be the best compromise with respect to transduction efficiency, cell viability and osteogenic effects. At an MOI of 100 using Ad.BMP-2, a highly significant increase in calcium deposition in monolayer hADSCs could be detected, while lower MOIs failed to stimulate calcium depositions significantly as compared to the MO group, indicating a threshold between an MOI of 50 and 100 in this hADSC monolayer culture. Our findings regarding the optimal MOI are in agreement with previous investigations of other scientists working with human processed lipoaspirate cells (PLAs) [39]. In this study by Dragoo et al., transduction of PLAs with Ad.BMP-2 at an MOI of 100 was also found to be optimal, resulting in high BMP-2 expression and low apoptosis. An MOI, the ratio of infectious viral particles to cells, can be determined for isolated cells but not for tissue fragments. Instead, according to our previous work, a dose of 4 × 10^8^ plaque-forming units (pfu) was used for transduction of round fragments of fascia tissue with a diameter of 4 mm. This dose has been identified to be optimal, leading to high transduction efficiency and a strong osteogenic response within the tissue fragments [31], which was confirmed in the present study.

In contrast to transduced hADSCs, gene-activated fascia produced lower but more uniform, constant levels of BMP-2 over 30 days. Despite considerably higher BMP-2 peak levels in the comparison groups, delivery by overexpressing fascia also led to a strong osteogenic response of hADSCs. Remarkably, there was no statistically significant difference with respect to ALP activity between BMP-2-transduced fascia as compared to BMP-2-transduced hADSCs or recombinant BMP-2 supplementation. Immunohistochemistry and histomorphometry revealed that hADSCs stimulated by BMP-2-transduced fascia produced significantly more OCN, OPN and Scl than those stimulated by recombinant BMP-2. In addition, regarding gene expression measured by quantitative RT-PCR, the same or even significantly higher levels of investigated bone markers were induced by BMP-2 gene-activated fascia than by recombinant BMP-2. However, the strongest induction of bone-related markers, at the gene and protein level, was noted for the group of BMP-2-transduced hADSCs, which was likely due to larger amounts of BMP-2 production. Hence, the results of the present study show that a constant level of BMP-2 as created by gene transfer induces a stronger osteogenic response in hADSCs than the repeated addition of similar or even higher amounts of recombinant protein. In addition, our experiments demonstrate that this superior effect on hADSCs can not only be achieved by direct genetic modification of hADSCs but also by BMP-2 delivery to hADSCs via transduced fascia tissue.

Fascia is a sheet of connective tissue consisting primarily of collagen with a large number of fibroblasts residing on the surface [31]. Previously, we have demonstrated that these fibroblasts can be readily transduced using adenoviral vectors, determining 4 × 10^8^ pfu as the optimal dose [31,33]. The harvest and transplantation of fascia alone or in combination with muscle is a common procedure frequently performed by plastic and reconstructive surgeons in order to cover soft tissue defects and reconstruct deformities [40,41]. Fascia has favorable physical properties as compared to muscle or adipose tissue, as it is resistant to tensile forces. Its tear resistance allows for the suturing of fascia to anatomical structures or the attachment of it to cell-seeded scaffolds.

In this study, we used a highly porous, biomimetic biphasic calcium-phosphate 3D scaffold with spongiosa-like morphology as a biomaterial because it combines a promising osteogenic capacity, suitable mechanical properties and biocompatibility, providing a controllable and biomimetic environment to study the effects of transient or continuous secretion of growth factors on stem cells. Tissue engineering of bone often involves cell seeding and growth factors delivered on scaffold materials. The feasibility of using hydroxyapatite (HA), β-tricalciumphosphate (β-TCP) and a mixture of both (BCP, biphasic calcium phosphate) to produce osteoinductive, resorbable scaffolds in combination with multipotential stem cells for bone repair ex vivo has been widely demonstrated. Our group uses scaffolds that have been developed as spongiosa-like synthetic bone substitute material leading to improved bone regeneration both in a mouse [42,43] and in a cranial minipig model [42]. Similar structures have already been used clinically, both on an experimental base [44] and as established commercial products. The modification used here features an extremely high open porosity at good mechanical stability while offering trabecular architecture very similar to spongious bone. A special feature is the triangular-shaped trabeculae that convex toward the junctions. This geometry is very similar to spongious bone trabeculae, promoting a natural cell morphology and formation of the cell skeleton. In summary, the biomimetic scaffold utilized in the present work imitates natural cancellous bone regarding material, stability and structure.

We used BMP-2 as a growth factor stimulating differentiation of hADSCs towards the osteogenic lineage since BMP-2 has been approved clinically [45] and its strong osteogenic effects on precursor cells are well-documented [46,47]. Recombinant BMP-2 has been applied in animal models [48,49] and patients [50,51] to enhance bone growth. However, the current delivery modes of these recombinant proteins are suboptimal due to their short half-life and side effects resulting from high dosage and poor local protein retention [52,53,54,55]. Therefore, various novel strategies for BMP-2 delivery are being investigated. For example, Bedair et al. developed a poly(lactide-co-glycolide) (PLGA) composite incorporated with magnesium hydroxide (MH) nanoparticles for improved BMP-2 delivery. Release of the growth factor proteins from their innovative carriers promoted cell proliferation and osteogenic differentiation in vitro as well as bone formation in a posterolateral spinal fusion model [56]. Another interesting approach is the use of gene-activated scaffolds. A report from Raftery et al. demonstrated that an optimized BMP-2 plasmid incorporated in a collagen-hydroxyapatite scaffold induced a strong osteogenic response in mesenchymal stem cells and accelerated bone formation in vivo [57]. Interestingly, in a recent study, osteogenic transdifferentiation of human umbilical cord vein endothelial cells (HUVECs) was achieved by transfection with the octamer-binding transcription factor 4 (OCT-4) gene followed by BMP-4 treatment. Implantation of a gelatin-heparin cryogel laden with the OCT-4-transfected and BMP-4-treated HUVECs led to enhanced repair of cranial defects. The authors presented a promising biomaterial and a single-cell source with high osteogenic potential [58].

In addition to previous work, the present study indicates that gene transfer could be a potential solution to the current clinical delivery problem [27,32]. However, there are two drawbacks associated with traditional ex vivo regional gene therapies, such as using BMP-2-transduced hADSCs seeded on scaffolds: (1) safety: isolated cells distributed on a biomaterial just briefly prior to implantation are more likely to become dislocated from the scaffold and may potentially lead to heterotopic bone formation; (2) translation: the genetic modification of isolated hADSCs with high efficiency requires more time than provided during a single surgical procedure. Therefore, we believe that the delivery of BMP-2 to hADSCs by overexpressing fascia may be an interesting alternative strategy enabling the development of an expedited ex vivo gene transfer approach with the potential for clinical translation. The present data encourage the evaluation of the approach’s safety profile and effectiveness for bone regeneration in vivo.

## 4. Materials and Methods

### 4.1. Study Design

This study was designed to investigate the osteogenic performance of human adipose-derived stem cells (hADSCs) on highly porous three-dimensional (3D) scaffolds with spongiosa-like morphology and to compare the osteoinductive effects of human BMP-2 (hBMP-2) delivered to hADSCs via different approaches—a direct and an indirect gene transduction approach and recombinant hBMP-2 supplementation. Human BMP-2 synthesis level in Ad.hBMP-2-transduced hADSC-seeded scaffold (the MO + Ad.hBMP-2 hADSC group) was monitored. The same concentration profile of recombinant hBMP-2 was provided to non-transduced hADSC-seeded scaffolds (the MO + rhBMP-2 group) by supplementing the recombinant growth factors to the culture medium. Another group of non-transduced hADSC-seeded scaffolds was co-cultured with Ad.hBMP-2-transduced muscle/fascia tissue fragments (the MO + Ad.hBMP-2 muscle group). Non-transduced hADSC-seeded scaffolds cultured in normal growth medium (the MN group) or osteogenic medium (the MO group) were analyzed in comparison to the above BMP-2-treated scaffolds.

The monolayer experiment was firstly investigated to explore the transduction efficiency to hADSCs using different multiplicities of infection (MOI). Meanwhile, toxic effect and osteogenic differentiation were evaluated in order to find a balance between safety and efficiency of virus transduction. The number of 4 × 10^8^ infectious units (IU) used for the transduction of muscle/fascia tissue fragments was decided based on results of our previous studies [31,33].

### 4.2. Monolayer Experiments

#### 4.2.1. Cell Culture

Human adipose-derived stem cells (hADSCs) were extracted from human subcutaneous fat, as previously reported [59,60]. Samples of human subcutaneous fat were provided by the biobank of the author´s institution. The established primary hADSCs were cultured in T75 culture flasks (Thermo Fischer Scientific, Waltham, MA, USA) with fresh growth medium (Dulbecco’s modified eagle’s medium (DMEM) (Gibco, Waltham, MA, USA), 15% fetal calf serum (FCS) (Sigma-Aldrich, Taufkirchen, Germany) and 60 IU/mL penicillin/streptomycin (Sigma-Aldrich, Taufkirchen, Germany) in a Binder FED 115 incubator (BINDER, Tuttlingen, Germany) (set at 37 °C/5% CO_2_). Media were changed every 3 days. The adherent cells were trypsinized and replated after reaching approximately 80% confluence. Passage 3 (P3) hADSCs were used for this study.

#### 4.2.2. Adenoviral Transduction

Passage 3 hADSCs harvested from T75 culture flasks (Thermo Fischer Scientific) were seeded on 24-well culture plates (Thermo Fischer Scientific, Waltham, MA, USA), with 2 × 10^4^ cells on each well. Cells were transduced with adenovirus-encoding hBMP-2 (Ad.hBMP-2) or green fluorescent protein (Ad.GFP) (Sirion Biotech, Martinsried, Germany) when reaching 70–80% confluency. hADSCs transduced with Ad.GFP were investigated for transduction efficiency, while Ad.hBMP-2-transduced hADSCs were employed for osteogenic differentiation analysis. Ad.hBMP-2 or Ad.GFP vectors were previously diluted at designed MOI of 0, 10, 20, 50, 100, 200 and 1000 in 1× PBS (Phosphate Buffered Saline) (Thermo Fischer Scientific, Waltham, MA, USA) and then added to the wells. The amount of PBS was enough to cover the whole layer of cells to prevent the cells from drying out. The transduced cells were then incubated in a Binder FED 115 incubator (BINDER) for 1 h and carefully rotated every 20 min. Cells were subsequently washed three times with 1× PBS, and fresh growth medium was added. For the osteogenic differentiation analysis, hADSCs were cultured with osteogenic medium.

#### 4.2.3. Fluorescence Activated Cell Sorting (FACS)

Twenty-four hours after transduction, the transduced hADSCs of each group were trypsinized and collected in 1× PBS for transduction efficiency analysis. Fluorescence-activated cell sorting (FACS) separated the hADSCs into two sub-populations depending on whether the cells were labeled with GFP. After being “interrogated” by the laser, each individual hADSC entered a single droplet as it left the nozzle tip. A single GFP-transduced hADSC in a single droplet were given a positive charge and be attracted to the corresponding collection tube, while non-transduced hADSCs were attracted to the other collection tube. Sorted cell populations were analyzed to calculate the percentage of GFP-transduced hADSCs in the total cell populations.

#### 4.2.4. Osteogenic Differentiation of hADSCs in Monolayer

Ad.hBMP-2-transduced hADSCs at MOI of 10, 20, 50, 100, 200 and 1000 were cultured with osteogenic medium on 24-well culture plates (2 × 10^4^ cells/well). The composition of the osteogenic medium was DMEM/Ham’s F-12 (Thermo Fischer Scientific) with 10% fetal calf serum (FCS) (Sigma-Aldrich), 50 μM L-ascorbic acid 2-phosphate (Sigma-Aldrich), 10 nM glycerophosphate (Sigma-Aldrich) and 10 nM dexamethasone (Sigma-Aldrich). Non-transduced hADSCs cultured in osteogenic medium (MO) or normal growth medium (MN) were set as controls. Alizarin red S staining was applied to detect calcium deposition. After 30 days of incubation, cells were fixed with ice cold 4% PFA solution (Thermo Fischer Scientific) and stained with 40 mM Alizarin red S (Sigma–Aldrich, St. Louis, MO, USA) solution (pH 4.1) for 10 min at RT and finally washed with PBS. Images were captured before and after staining, respectively, using an M8 Microscope and Scanner (PreciPoint, Freising, Germany) with ViewPoint Scanner software (VuPoint Solutions, City of Industry, CA, USA). Quantitative analysis was performed utilizing Image J software v.1.6 (NIH, Bethesda, MD, USA, https://imagej.nih.gov). The positively Alizarin red S-stained area and the total area of each well were calculated, respectively. At least nine samples per group were used for the quantitative analysis. Values were expressed as a mean percentage of the positive part within each sample.

#### 4.2.5. Water-Soluble Tetrazolium-1 (WST-1) Assay

After 1, 2, 3 and 4 weeks of culture, WST-1 (Roche, Mannheim, Germany) assay was applied to evaluate the proliferation of hADSCs transduced with Ad.hBMP-2 using different MOI. Briefly, culture medium mixed with WST-1 at a ratio of 10:1 was prepared. The culture medium in the well was subsequently replaced by the medium-WST-1 mixture and incubated together with the samples for 2 h in the incubator (BINDER). The absorbance at 450 nm was measured using a Synergy HT microplate reader (BioTek, Bad Friedrichshall, Germany) with Gen 5 2.03 software (BioTek, Bad Friedrichshall, Germany).

### 4.3. Three-Dimensional (3D) Scaffold Experiments

#### 4.3.1. Fabrication of Scaffolds

RePore Sponge ceramic scaffolds are a standard 3D-culture reference offered by BioMed Center Innovation gGmbH, Bayreuth, Germany. The scaffolds were produced by a modified foam-casting technique. Polyurethane packaging foams with 35 ppi pore distribution were activated by consecutive treatment in dimethylene-diisocyanate for 1 h and polyethylene-glycol solution for 2–3 h. Acetone was used as solvent for both treatments and as washing solution. PEG-coated scaffolds were dipped into a slurry with 65 wt% BCP-powder, which had been prepared before from 60 wt% HA and 40 wt% β-TCP, both calcined at 900 °C by 24 h mixing in a tumble-mixer (Turbula). The composition of the ceramic slurry, containing approx. 5 wt% organic additives, is proprietary. Dipped scaffolds were freed from surplus slurry by air pressure until all pores were open, then left to dry overnight at 70 °C and coated again with the same procedure. After the second drying step, scaffolds were sintered for 1 h at 1250 °C, with a holding step at 600 °C to incinerate the organic PU-foam support. The sintered scaffolds are free of carbon compounds (determined in previous investigations). They attain a porosity of 68.0 ± 2.1% as determined from absolute volume fraction compared to geometric volume and have a material density (skeletal density) of 2.964 ± 0.006 g/cm³, as measured by helium pycnometry (AccuPyc, Micromeritics, Unterschleissheim, Germany)).

#### 4.3.2. hADSC Colonization on Scaffolds

Previously sterilized scaffolds were transferred to 12-well culture plates (Thermo Fischer Scientific, Waltham, MA, USA) (one scaffold per well) with a 6 h incubation in cell culture medium before colonization to buffer possible pH reactants and humidify scaffolds. Harvested passage 3 hADSCs were suspended in fresh growth medium at a concentration of 5.0 × 10^6^ cells/mL. Each scaffold was evenly seeded by 200 μL aliquot cell suspension and then stored in the incubator (BINDER) for 20 min to allow the cells to attach. Subsequently, 1.5 mL of normal growth medium was added carefully to each well for 24 h of incubation. Significantly, for the MO + Ad.hBMP-2 hADSC group, 24 h prior to scaffold seeding, passage 3 hADSCs cultured in the flasks were transduced with Ad.hBMP-2 at the MOI of 100. An MOI of 100 was chosen based on the evidence of transduction efficiency and calcium deposition from monolayer experiments.

#### 4.3.3. Harvest of Muscle/Fascia Tissue and Transduction with Adenoviral Vectors

Muscle tissue with attached fascia was harvested from the hind limbs of three donor Fischer rats (F-344/DuCrl) (Charles River, Sulzfeld, Germany). With 4 mm dermal biopsy punches (pfm medical, Cologne, Germany), round muscle/fascia tissue fragments were created and transferred to 12-well plates (5 pieces per well). Subsequently, 4 × 10^8^ infectious units (IU) of Ad.GFP (Sirion Biotech) or Ad.hBMP-2 (Sirion Biotech) in 10 μL vector solution were evenly pipetted to the surface of the fascia layer of each tissue fragment, followed by 1 h incubation in the incubator (BINDER). Fresh growth media were added after transduction. To detect the transduction efficiency, 24 h after transduction, unbound vector particles were removed by rinsing with PBS. Pictures were taken using an LSM 880 laser-scanning confocal microscope (Zeiss, Jena, Germany). Non-transduced tissue discs were analyzed as a control.

#### 4.3.4. Culture Conditions

Twenty-four hours after cell seeding, the scaffolds were transferred into new 12-well culture plates (Thermo Fischer Scientific) with 1.5 ml of osteogenic medium. Medium was collected every 3 days for a period of 4 weeks. Regarding the MO + rhBMP-2 group, recombinant hBMP-2 was added to the osteogenic medium at the exact concentrations that had been previously detected to be synthesized by the MO + Ad.hBMP-2 hADSC group over a period of 4 weeks. For the MO + Ad.hBMP-2 fascia group, non-transduced hADSC-seeded scaffolds were co-cultured with Ad.hBMP-2-transduced muscle/fascia fragments (1 scaffold + 5 muscle/fascia-fragments/well). A circular plastic bracket was designed to support the scaffold and avoid direct contact with the underlying muscle/fascia fragments. The bracket was created in each well to keep the same culture environment for all the groups.

#### 4.3.5. Detection of hBMP-2 Levels by Enzyme-Linked Immunosorbent Assay (ELISA)

Medium was collected from each well every 3 days. Samples from MN and MO groups were set as controls. Enzyme-linked immunosorbent assay (ELISA) (Bio-Techne, Wiesbaden-Nordenstadt, Germany) was applied to quantify the protein level in each group according to the manufacturer’s instructions. The absorbance was measured at 450 nm wavelength using a Synergy HT microplate reader (BioTek). A standard curve was created based on the absorbance of the corresponding protein concentration. Each measurement was performed in triplicate. Nine samples per group were used for the assay.

#### 4.3.6. H&E Staining of hADSC-Seeded Scaffolds

After 28 days of incubation, hADSC-seeded scaffold constructs were washed 3 times with PBS and fixed in acetone for 3 min. Subsequently, scaffolds were stained with haematoxylin (Sigma-Aldrich, St. Louis, MO, USA) for 10 min, followed by a thorough rinse in running tap water and then stained with eosin (Sima-Aldrich) for 3 min. After dehydration in 100% ethanol, the scaffolds were air-dried at room temperature. Images were taken using a VHX-5000 3D digital microscope (Keyence, Osaka, Japan) equipped with VHX-5000 Ver. 1.6.1.0/System Ver. 1.04 software (Keyence, Osaka, Japan).

#### 4.3.7. Alkaline Phosphatase (ALP) Activity Determination

The scaffolds of each group were harvested after 1, 2 and 4 weeks of culture, and ALP activity was determined using an ALP enzyme kit (Abcam, Cambridge, UK). The absorbance was measured on a Synergy HT microplate reader (BioTek) at 400 nm wavelength. The standard curve was firstly created following the manufacturer’s instructions. ALP activity was normalized by DNA contents as quantified by PicoGreen dsDNA assay kit (Thermo Fisher Scientific, Waltham, MA, USA).

#### 4.3.8. Quantitative Reverse Transcriptase Polymerase Chain Reaction (qRT-PCR)

After 1, 2 and 4 weeks of incubation, the scaffolds were homogenized with a Mikro-Dismembrator S (Sartorius, Goettingen, Germany). Total mRNA was extracted using TRIzol reagent (Qiagen, Hilden, Germany) and reverse-transcribed into cDNA using the QuantiTect Reverse Transcription-Kit (Qiagen, Hilden, Germany). One μg was used to perform the reverse transcription.

Alkaline phosphatase (ALP), Runt-related transcription factor 2 (RUNX-2), Osteopontin (OPN), Bone Sialoprotein (BSP), Osteocalcin (OCN), human Bone Morphogenetic Protein-2 (hBMP-2) and the housekeeping gene’s glyceraldehyde-3-phosphate-dehydrogenase (GAPDH) expression were quantified using FastStart Essential DNA Green Master (Roche Applied Science, Penzberg, Germany) on the Light Cycler 96 thermocycler (Roche Diagnostics, Penzberg, Germany). PCR reaction conditions were a denaturation step of 95 °C for 1 min, 40 cycles of 95 °C for 10 s, 60 °C for 15 s and 72 °C for 15 s and a final extension step at 37 °C for 5 min. The relative gene expression was calculated using the 2^−ΔΔCt^ method. The data were normalized to untreated passage 3 hADSCs.

#### 4.3.9. Mineralization Detection in the Co-Cultured Muscle/Fascia Fragments

After 4 weeks of incubation together with hADSC-seeded scaffolds, Ad.hBMP-2-transduced muscle/fascia fragments were harvested for calcification analysis. The tissue fragments were firstly fixed in 4% paraformaldehyde (PFA) (Thermo Fischer Scientific) for 24 h and then processed with a tissue processor, STP-120 (Thomas-medical, Mitterhofen, Austria), for fixation and paraffin infiltration. After embedding in paraffin blocks, fragments were cut at 8 µm with a Leica RM2255 microtome (Leica, Wetzlar, Germany). Mineralization in the tissue fragments was detected by Alizarin red S staining. After fixing with 4% paraformaldehyde (PFA) (Thermo Fischer Scientific) for 15 min, sections were treated with 40 mM Alizarin red S (Sigma-Aldrich) (PH 4.1) for 10 min. Thereafter, sections were dehydrated and air-dried at room temperature for 24 h. Calcium deposition was detected under an M8 Microscope (PreciPoint) with ViewPoint Scanner software (VuPoint Solutions).

#### 4.3.10. Immunofluorescent Staining of hADSC-Seeded Scaffolds

For 24 h, hADSC-seeded scaffold constructs were fixed in 4% PFA (Thermo Fischer Scientific) at 4 °C, then embedded in 1% agarose solution (Sakura, Tokyo, Japan). All the scaffolds were decalcified in 15% EDTA solution (DCS Innovative, Hamburg, Germany) for 3 weeks, then embedded in paraffin. Scaffolds were cut at 10 µm with a Leica RM2255 microtome (Leica). Tissue sections were fixed with acetone (Applichem, Darmstadt, Germany) for 15 min, blocked with 5% bovine serum albumin (BSA) (Sigma-Aldrich) for 30 min and permeabilized with 0.1% Triton X-100 solution (Sigma-Aldrich) in PBS (5 min). Samples were incubated first with primary antibody for 1 h at 4 °C and then with 1:500 diluted secondary antibody for 30 min at room temperature. The primary antibody was omitted for negative control. The primary antibodies (OCN, OPN, Scl) were purchased from R&D Systems (Wiesbaden, Germany) and the secondary antibody conjugated with Alexa Fluor 488 was obtained from Thermo Fischer Scientific. Cell nuclei were stained with Hoechst 33,342 (Sigma-Aldrich ) and then air-dried in darkness at 4 °C. The staining results were examined under a Zeiss Axioskop 40 (Zeiss, Jena, Germany).

#### 4.3.11. Histomorphometry

Quantitative analysis of the stained sections was investigated using Image J software v.1.6 (NIH, Bethesda, MD, USA). Nine samples per group were used for histomorphometry. The positive stained area and total area of each section were established, respectively. The mean percentage of positive stained area within the sections was indicated for the comparison.

### 4.4. Statistical Analysis

Nine samples per group were used for each assay and the measurements were performed in triplicate. Data were presented as mean ± standard error. Statistical analyses were performed using GraphPad Prism software (GraphPad Software, San Diego, CA, USA). Data were evaluated with an unpaired Student’s *t*-test for two-group comparisons and a Kruskal–Wallis test for comparing more than 2 groups. Comparisons with p values of < 0.05 were considered statistically different. Statistical significance was indicated by * for *p* < 0.05, ** for *p* < 0.01 and *** for *p* < 0.001.

## 5. Conclusions

Based upon the results of the present study, we conclude that fascia transduced by adenoviral vectors can serve as a delivery system for BMP-2-inducing osteogenic differentiation of hADSCs seeded on biomimetic scaffolds. This work may contribute to the development of an expedited, single-surgery method for bone repair, providing stem cells, a scaffold and sustained delivery of osteogenic molecules. Further studies are needed to investigate the effectiveness of the proposed bone regeneration strategy in vivo.

## Data Availability

The data presented in this study are available on request from the corresponding author.

## References

[1] Heiple K.G., Chase S.W., Herndon C.H. (1964). A Comparative Study of the Healing Process Following Different Types of Bone Transplantation. Plast. Reconstr. Surg..

[2] Bauer T.W., Muschler G.F. (2000). Bone Graft Materials: An Overview of the Basic Science. Clin. Orthop. Relat. Res..

[3] Control C.F.D. (1988). Transmission of HIV Through Bone Transplantation: Case Report and Public Health Recommendations. MMWR. Morb. Mortal. Wkly. Rep..

[4] Stevenson S. (1987). The immune response to osteochondral allografts in dogs. J. Bone Jt. Surg..

[5] Gurdon J.B., Bourillot P.Y. (2001). Morphogen gradient interpretation. Nature.

[6] Hajimiri M., Shahverdi S., Kamalinia G., Dinarvand R. (2015). Growth factor conjugation: Strategies and applications. J. Biomed. Mater. Res. Part A.

[7] Henkel J., Woodruff M.A., Epari D.R., Steck R., Glatt V., Dickinson I.C., Choong P.F., Schuetz M.A., Hutmacher D.W. (2013). Bone regeneration based on tissue engineering conceptions—A 21st century perspective. Bone Res..

[8] Silva A.K.A., Richard C., Bessodes M., Scherman D., Merten O.-W. (2008). Growth Factor Delivery Approaches in Hydrogels. Biomacromolecules.

[9] Taekhee J., Hwan L.J., Soonjung P., Yong-Jin K., Joseph S., Hye-Eun S., Ki-Suk K., Hyon-Seok J., Hyung-Min C., Seong-Geun O. (2017). Effect of BMP-2 Delivery Mode on Osteogenic Differentiation of Stem Cells. Stem Cells Int..

[10] Woo E.J. (2012). Recombinant human bone morphogenetic protein-2: Adverse events reported to the Manufacturer and User Facility Device Experience database. Spine J..

[11] Tessmar J.K., Göpferich A.M. (2007). Matrices and scaffolds for protein delivery in tissue engineering. Adv. Drug Deliv. Rev..

[12] Chung H.J., Park T.G. (2007). Surface engineered and drug releasing pre-fabricated scaffolds for tissue engineering. Adv. Drug Deliv. Rev..

[13] Pakulska M.M., Miersch S., Shoichet M.S. (2016). Designer protein delivery: From natural to engineered affinity-controlled release systems. Science.

[14] Sheridan M.H., Shea L.D., Peters M.C., Mooney D.J. (2000). Bioabsorbable polymer scaffolds for tissue engineering capable of sustained growth factor delivery. J. Control. Release.

[15] Atasoy-Zeybek A., Kose G.T. (2018). Gene Therapy Strategies in Bone Tissue Engineering and Current Clinical Applications. Adv. Exp. Med. Biol..

[16] Betz V.M., Kochanek S., Rammelt S., Müller P.E., Betz O.B., Messmer C. (2018). Recent advances in gene-enhanced bone tissue engineering. J. Gene Med..

[17] Bondarava M., Cattaneo C., Ren B., Thasler W.E., Jansson V., Müller P.E., Betz O.B. (2017). Osseous differentiation of human fat tissue grafts: From tissue engineering to tissue differentiation. Sci. Rep..

[18] Zuk P.A., Zhu M., Ashjian P., De Ugarte D.A., Huang J.I., Mizuno H., Alfonso Z.C., Fraser J.K., Benhaim P., Hedrick M.H. (2002). Human adipose tissue is a source of multipotent stem cells. Mol. Biol. Cell.

[19] Mohamed-Ahmed S., Fristad I., Lie S.A., Suliman S., Mustafa K., Vindenes H., Idris S.B. (2018). Adipose-derived and bone marrow mesenchymal stem cells: A donor-matched comparison. Stem Cell Res. Ther..

[20] Vakhshori V., Bougioukli S., Sugiyama O., Kang H.P., Tang A.H., Park S.H., Lieberman J.R. (2020). Ex vivo regional gene therapy with human adipose-derived stem cells for bone repair. Bone.

[21] Peterson B., Zhang J., Iglesias R., Kabo M., Hedrick M., Benhaim P., Lieberman J.R. (2005). Healing of critically sized femoral defects, using genetically modified mesenchymal stem cells from human adipose tissue. Tissue Eng..

[22] Davy P.M., Lye K.D., Mathews J., Owens J.B., Chow A.Y., Wong L., Moisyadi S., Allsopp R.C. (2015). Human adipose stem cell and ASC-derived cardiac progenitor cellular therapy improves outcomes in a murine model of myocardial infarction. Stem Cells Cloning Adv. Appl..

[23] Betz O.B., Betz V.M., Abdulazim A., Penzkofer R., Schmitt B., Schröder C., Augat P., Jansson V., Müller P.E. (2009). Healing of large segmental bone defects induced by expedited bone morphogenetic protein-2 gene-activated, syngeneic muscle grafts. Hum. Gene Ther..

[24] Betz O.B., Betz V.M., Abdulazim A., Penzkofer R., Schmitt B., Schröder C., Mayer-Wagner S., Augat P., Jansson V., Müller P.E. (2010). The repair of critical-sized bone defects using expedited, autologous BMP-2 gene-activated fat implants. Tissue Eng. Part A.

[25] Betz O.B., Betz V.M., Schröder C., Penzkofer R., Göttlinger M., Mayer-Wagner S., Augat P., Jansson V., Müller P.E. (2013). Repair of large segmental bone defects: BMP-2 gene activated muscle grafts vs. autologous bone grafting. BMC Biotechnol..

[26] Betz V.M., Betz O.B., Rosin T., Keller A., Thirion C., Salomon M., Manthey S., Augat P., Jansson V., Müller P.E. (2015). The effect of BMP-7 gene activated muscle tissue implants on the repair of large segmental bone defects. Injury.

[27] Betz V.M., Betz O.B., Rosin T., Keller A., Thirion C., Salomon M., Manthey S., Augat P., Jansson V., Müller P.E. (2016). An expedited approach for sustained delivery of bone morphogenetic protein-7 to bone defects using gene activated fragments of subcutaneous fat. J. Gene Med..

[28] Betz V.M., Keller A., Foehr P., Thirion C., Salomon M., Rammelt S., Zwipp H., Burgkart R., Jansson V., Müller P.E. (2017). BMP-2 gene activated muscle tissue fragments for osteochondral defect regeneration in the rabbit knee. J. Gene Med..

[29] Betz V.M., Ren B., Messmer C., Jansson V., Betz O.B., Müller P.E. (2018). Bone morphogenetic protein-2 is a stronger inducer of osteogenesis within muscle tissue than heterodimeric bone morphogenetic protein-2/6 and -2/7: Implications for expedited gene-enhanced bone repair. J. Gene Med..

[30] Betz V.M., Ren B., Betz O.B., Jansson V., Müller P.E. (2021). Osteoinduction within adipose tissue fragments by heterodimeric bone morphogenetic Proteins-2/6 and -2/7 versus homodimeric bone morphogenetic protein-2: Therapeutic implications for bone regeneration. J. Gene Med..

[31] Ren B., Betz V.M., Thirion C., Salomon M., Jansson V., Müller P.E., Betz O.B. (2018). Gene-activated tissue grafts for sustained bone morphogenetic protein-2 delivery and bone engineering: Is muscle with fascia superior to muscle and fat?. J. Tissue Eng. Regen. Med..

[32] Ren B., Betz V.M., Thirion C., Salomon M., Klar R.M., Jansson V., Müller P.E., Betz O.B. (2019). Gene activated adipose tissue fragments as advanced autologous biomaterials for bone regeneration: Osteogenic differentiation within the tissue and implications for clinical translation. Sci. Rep..

[33] Ren B., Betz V.M., Thirion C., Salomon M., Jansson V., Müller P.E., Betz O.B. (2019). Osteoinduction within BMP-2 transduced muscle tissue fragments with and without a fascia layer: Implications for bone tissue engineering. Gene Ther..

[34] Zügel M., Maganaris C.N., Wilke J., Jurkat-Rott K., Klingler W., Wearing S.C., Findley T., Barbe M.F., Steinacker J.M., Vleeming A. (2018). Fascial tissue research in sports medicine: From molecules to tissue adaptation, injury and diagnostics: Consensus statement. Br. J. Sports Med..

[35] Blasi M., Blasi J., Domingo T., Pérez-Bellmunt A., Miguel-Pérez M. (2015). Anatomical and histological study of human deep fasciae development. Surg. Radiol. Anat. SRA.

[36] Stecco C., Macchi V., Porzionato A., Duparc F., De Caro R. (2011). The fascia: The forgotten structure. Ital. J. Anat. Embryol..

[37] Betz V.M., Betz O.B., Harris M.B., Vrahas M.S., Evans C.H. (2008). Bone tissue engineering and repair by gene therapy. Front. Biosci. J. Virtual Libr..

[38] Durham H.D., Lochmüller H., Jani A., Acsadi G., Massie B., Karpati G. (1996). Toxicity of replication-defective adenoviral recombinants in dissociated cultures of nervous tissue. Exp. Neurol..

[39] Dragoo J.L., Choi J.Y., Lieberman J.R., Huang J., Zuk P.A., Zhang J., Hedrick M.H., Benhaim P. (2003). Bone induction by BMP-2 transduced stem cells derived from human fat. J. Orthop. Res..

[40] Veyrat M., Verillaud B., Herman P., Bresson D. (2016). How I do it. The pedicled temporoparietal fascia flap for skull base reconstruction after endonasal endoscopic approaches. Acta Neurochir..

[41] Nuri T., Ueda K., Yamada A. (2017). Application of free serratus anterior fascial flap for reconstruction of ear deformity due to hemifacial microsomia: A report of two cases. Microsurgery.

[42] Roldán J.C., Schulz P., Klünter T., Deisinger U., Diez C., Waiss W., Kirschneck C., Reichert T.E., Detsch R. (2017). BMP-7 Preserves Surface Integrity of Degradable-ceramic Cranioplasty in a Göttingen Minipig Model. Plast. Reconstr. Surg. Glob. Open.

[43] Roldán J.C.D.R., Schaefer S., Chang E., Kelantan M., Waiss W., Reichert T.E., Gurtner G.C., Deisinger U. (2010). Bone formation and degradation of a highly porous biphasic calcium phosphate ceramic in presence of BMP-7, VEGF and mesenchymal stem cells in an ectopic mouse model. J. Craniomaxillofac. Surg..

[44] Scarano A., Lorusso F., Santos de Oliveira P., Kunjalukkal Padmanabhan S., Licciulli A. (2019). Hydroxyapatite Block Produced by Sponge Replica Method: Mechanical, Clinical and Histologic Observations. Materials.

[45] Razzouk S., Sarkis R. (2012). BMP-2: Biological challenges to its clinical use. New York State Dent. J..

[46] Zhou N., Li Q., Lin X., Hu N., Liao J.Y., Lin L.B., Zhao C., Hu Z.M., Liang X., Xu W. (2016). BMP2 induces chondrogenic differentiation, osteogenic differentiation and endochondral ossification in stem cells. Cell Tissue Res..

[47] Song I., Kim B.S., Kim C.S., Im G.I. (2011). Effects of BMP-2 and vitamin D3 on the osteogenic differentiation of adipose stem cells. Biochem. Biophys. Res. Commun..

[48] Cleemann R., Sorensen M., Bechtold J.E., Soballe K., Baas J. (2018). Healing in peri-implant gap with BMP-2 and systemic bisphosphonate is dependent on BMP-2 dose-A canine study. J. Orthop. Res..

[49] Kaipel M., Schützenberger S., Schultz A., Ferguson J., Slezak P., Morton T.J., Van Griensven M., Redl H. (2012). BMP-2 but not VEGF or PDGF in fibrin matrix supports bone healing in a delayed-union rat model. J. Orthop. Res..

[50] Kohan E., Roostaeian J., Yuan J.T., Fan K.L., Federico C., Kawamoto H., Bradley J.P. (2015). Customized bilaminar resorbable mesh with BMP-2 promotes cranial bone defect healing. Ann. Plast. Surg..

[51] Haubruck P., Tanner M.C., Vlachopoulos W., Hagelskamp S., Miska M., Ober J., Fischer C., Schmidmaier G. (2018). Comparison of the clinical effectiveness of Bone Morphogenic Protein (BMP) -2 and -7 in the adjunct treatment of lower limb nonunions. Orthop. Traumatol. Surg. Res. OTSR.

[52] Garrison K.R., Donell S., Ryder J., Shemilt I., Mugford M., Harvey I., Song F. (2007). Clinical effectiveness and cost-effectiveness of bone morphogenetic proteins in the non-healing of fractures and spinal fusion: A systematic review. Health Technol. Assess..

[53] Shields L.B., Raque G.H., Glassman S.D., Campbell M., Vitaz T., Harpring J., Shields C.B. (2006). Adverse effects associated with high-dose recombinant human bone morphogenetic protein-2 use in anterior cervical spine fusion. Spine.

[54] James A.W., LaChaud G., Shen J., Asatrian G., Nguyen V., Zhang X., Ting K., Soo C. (2016). A Review of the Clinical Side Effects of Bone Morphogenetic Protein-2. Tissue Eng. Part B Rev..

[55] Haidar Z.S., Hamdy R.C., Tabrizian M. (2009). Delivery of recombinant bone morphogenetic proteins for bone regeneration and repair. Part A: Current challenges in BMP delivery. Biotechnol. Lett..

[56] Bedair T.M., Lee C.K., Kim D.S., Baek S.W., Bedair H.M., Joshi H.P., Choi U.Y., Park K.H., Park W., Han I. (2020). Magnesium hydroxide-incorporated PLGA composite attenuates inflammation and promotes BMP2-induced bone formation in spinal fusion. J. Tissue Eng..

[57] Raftery R.M., Mencía-Castaño I., Sperger S., Chen G., Cavanagh B., Feichtinger G.A., Redl H., Hacobian A., O’Brien F.J. (2018). Delivery of the improved BMP-2-Advanced plasmid DNA within a gene-activated scaffold accelerates mesenchymal stem cell osteogenesis and critical size defect repair. J. Control. Release.

[58] Kim S.H.L., Lee S.S., Kim I., Kwon J., Kwon S., Bae T., Hur J., Lee H., Hwang N.S. (2020). Ectopic transient overexpression of OCT-4 facilitates BMP4-induced osteogenic transdifferentiation of human umbilical vein endothelial cells. J. Tissue Eng..

[59] Jiang T., Liu W., Lv X., Sun H., Zhang L., Liu Y., Zhang W.J., Cao Y., Zhou G. (2010). Potent in vitro chondrogenesis of CD105 enriched human adipose-derived stem cells. Biomaterials.

[60] Huang Y., Seitz D., König F., Müller P.E., Jansson V., Klar R.M. (2019). Induction of Articular Chondrogenesis by Chitosan/Hyaluronic-Acid-Based Biomimetic Matrices Using Human Adipose-Derived Stem Cells. Int. J. Mol. Sci..

